# Editorial: Redox signaling and homeostasis in the control of metabolism: a systemic interplay between central and peripheral effectors

**DOI:** 10.3389/fendo.2024.1522613

**Published:** 2024-12-03

**Authors:** Letizia Palomba, Cristoforo Silvestri, Elena Silvestri

**Affiliations:** ^1^ Institute of Biomolecular Chemistry, National Research Council of Italy, Pozzuoli, Italy; ^2^ Department of Biomolecular Sciences, University of Urbino Carlo Bo, Urbino, Italy; ^3^ Centre de Recherche de l’Institut de Cardiologie et Pneumologie de Quebéc (CRIUCPQ) et Institut sur la Nutrition et les Aliments Fonctionnels (INAF), Centre NUTRISS, Department of Medicine and School of Nutrition, Université Laval, Quebec, QC, Canada; ^4^ Department of Science and Technology, University of Sannio, Benevento, Italy

**Keywords:** oxidative stress, mitochondria, inflammation, obesity, non-alcoholic fatty liver disease, cardiovascular disease, type 2 diabetes

The relationship between redox signaling, homeostasis, and metabolic regulation has drawn considerable attention, especially in the context of chronic conditions such as obesity, diabetes, and cardiovascular disease (CVD) (1). This Research Topic comprising two original research articles, two mini-reviews, and one review, provides new insights into the interplay between oxidative stress, redox balance, and metabolic processes highlighting their significance for human health and disease mechanisms. Redox signaling and homeostasis involve balancing reactive species, including free radicals, which regulate cellular processes (2). However, an imbalance leading to oxidative stress contributes to the development of diseases such as non-alcoholic fatty liver disease (NAFLD), type 2 diabetes (T2D), and CVD. Despite advances in understanding the mechanisms of oxidative stress, challenges remain, particularly in the regulation of redox homeostasis within specific cellular environments. The five papers summarized here address these issues, providing new insights into the therapeutic potential of targeting redox imbalances in metabolic dysfunctions.

The original research article by Giacco et al. focuses on mitochondrial quality control (MQC) in hypothyroidism, a condition characterized by oxidative imbalance and inflammation. The study demonstrates that within an experimental hypothyroidism model, the iodothyronines T3 and 3,5-T2 help restore oxidative balance by increasing antioxidant defense systems within livers, reduce mitochondrial DNA damage, and lower inflammation. Notably, 3,5-T2 is more effective than T3 in alleviating inflammation via the cGAS-STING pathway, which is triggered by damage-associated molecular patterns (mtDAMPs), highlighting its potential therapeutic role in treating sterile inflammatory diseases such as NAFLD which is often associated with hypothyroidism.

The other original research article by Zhang et al. examines the role of NADPH in regulating ROS in periodontal ligament stem cells (PDLSCs) under diabetic conditions. Under conditions of high glucose to simulate diabetic hyperglycaemia, PDLSCs had excessive NADPH production and ROS accumulation and impaired osteogenic differentiation, a critical process for periodontal tissue regeneration that is impaired in people with diabetes. This work highlights potential new therapeutic approaches for diabetes-related cellular dysfunction by targeting NADPH and, when taken into consideration with the work presented by Giacco et al. demonstrates the breadth of biological systems in which redox signalling is critical for homeostatic regulation.

Additionally, the three collected reviews explore redox homeostasis in diseases associated with metabolic syndrome: T2D, CVD, and obesity.

In their analytical mini review Holendova and Plecita-Hlavata focus on cysteine modifications in pancreatic beta cells, essential for insulin secretion. While redox modifications are crucial for maintaining beta-cell function, excessive oxidative stress can cause irreversible damage, contributing to beta-cell failure and progression of T2D.

In their insightful and critical mini review, Zullo et al. examine the role of mitochondrial sirtuins (SIRT3, SIRT4, and SIRT5) in regulating oxidative stress and energy metabolism in CVD. These sirtuins modulate key processes such as autophagy and gene expression, highlighting their therapeutic potential for mitigating CVD, including atherosclerosis.

In their highly informative and comprehensive review, Franco and Canzoniero examine the role of zinc and redox homeostasis in obesity, They discuss how zinc-binding proteins and transporters regulate cellular zinc levels, affecting redox metabolism. Disrupted zinc homeostasis in obesity exacerbates oxidative stress, suggesting that zinc, the levels of which have been found to be low in obese people, plays a critical role in the pathogenesis of obesity by regulating redox homeostasis and they may be a potential therapeutic agent in metabolic diseases.

Collectively, all five papers emphasize the central role of oxidative stress and redox signaling in chronic metabolic diseases, addressing both systemic and localized mechanisms. Together, they underscore the therapeutic potential of modulating redox homeostasis across various metabolic disorders. These studies provide a comprehensive understanding of redox balance in metabolic health. Giacco et al.‘s research on iodothyronines opens new possibilities for treating inflammation in hypothyroidism by targeting specific molecular pathways. Likewise, the exploration of zinc transporters and sirtuins provides novel perspectives on the regulation of nutrients and enzymes in obesity and CVD. The role of sirtuins in mitochondrial pathways is particularly promising, with research suggesting their potential to regulate oxidative stress and energy metabolism. Studies on SIRT3 and SIRT5 emphasize their importance in maintaining mitochondrial integrity and oxidative balance, particularly in the context of metabolic syndrome and aging (3, 4).

However, several challenges remain. While 3,5-T2 shows potential in reducing inflammation and restoring mitochondrial function, its long-term safety and efficacy in human clinical settings are still largely unexplored. Concerns about its translational potential arise due to metabolic differences between humans and the rodent models commonly used in these studies (5, 6). In the context of zinc and redox homeostasis, the relationship between systemic zinc regulation and obesity-induced oxidative stress requires further clarification. Zinc transporters, such as ZnT and ZIP, play crucial roles in maintaining homeostasis, but more research is needed to fully understand how these pathways are modulated under different metabolic conditions (7). In Yin et al.’s study on NADPH and ROS, the therapeutic potential of modulating NADPH production still needs further investigation. While NADPH is essential for antioxidant defenses, it can also contribute to metabolic imbalances, making therapeutic targeting complex (8). Finally, the review on cysteine modifications in beta cells highlights the need for more research into the interaction between lipid metabolism and redox signaling in beta-cell function. Although cysteine modifications are well studied, their specific roles in the pathology of diabetes remain an area of active investigation (9).

These studies have important implications for both basic science and clinical practice. Understanding how redox signaling influences metabolic diseases could pave the way for new therapeutic strategies, such as targeting mitochondrial sirtuins in CVD or modulating zinc homeostasis in obesity. The role of redox regulation in stem cell differentiation also opens promising avenues for tissue regeneration therapies. Future research should aim to unravel the complex crosstalk between redox signaling, mitochondrial function, and metabolic pathways across different tissues. More in-depth studies are needed to explore how therapeutic interventions targeting redox homeostasis can be safely and effectively implemented in chronic disease management. Moreover, understanding individual variability and genetic predispositions to oxidative stress will be crucial for developing personalized treatments. This Research Topic highlights the central role of redox signaling and homeostasis in metabolic regulation and chronic disease. From mitochondrial dysfunction in hypothyroidism to sirtuin regulation in CVD, these papers underline the complexity and therapeutic potential of targeting oxidative stress mechanisms. Continued research on the interplay between redox signaling, metabolism, and chronic disease will be essential for developing more effective treatments to combat the growing burden of metabolic disorders.
